# Invalid and Crippled Children

**Published:** 1920-08-21

**Authors:** 


### Invalid and Crippled Children.

A joint conference on "Work for Invalid and Crippled Children" is to be held by the Invalid Children's Aid Association and the Central Committee for the Care of Cripples on November 1[illegible] and 17 at the Guildhall, London. The subjects for discussion include the bearing of recent legislation on work for physically defective children; co-operation between the State and voluntary bodies; accounts of existing institutions in America, on the Continent, and in the United Kingdom for the treatment and education of these children; and the scheme for future development suggested by the Central Committee for the Care of Cripples. Further particulars may be obtained from the Conference Secretary, Central Committee for Care of Cripples, 20 Berkeley Street, London, W.

